# Characterization and Classification of Iranian Honey Based on Physicochemical Properties and Antioxidant Activities, with Chemometrics Approach

**Published:** 2018

**Authors:** Hamid Moloudian, Sepideh Abbasian, Nasser Nassiri-Koopaei, Mohammad Reza Tahmasbi, Golnaz alsadat Afzal, Maryam Sadat Ahosseini, Masud Yunesian, Mohammad Reza Khoshayand

**Affiliations:** a *Pharmaceutical Quality Assurance Research Center, Tehran University of Medical Sciences, Tehran, Iran.*; b *Department of Drug and Food Control, Faculty of Pharmacy, Tehran University of Medical Sciences, Tehran, Iran.*; c *Department of Environmental Health Engineering, School of Public Health, Tehran University of Medical Sciences, Tehran, Iran.*

**Keywords:** Antioxidant activity, Chemometrics, Classification, Physicochemical properties, Honey

## Abstract

In the present study, the physicochemical properties and antioxidant activities of different Iranian honey samples are investigated using various multivariate techniques in order to develop a quality control model. Forty-eight Iranian honey samples were tested for 15 physicochemical and antioxidant parameters. The parameters for which the samples were tested included color intensity, moisture, electrical conductivity, pH, free acidity, diastase activity, hydroxymethylfurfural content, proline level, total phenolic content, antioxidant activity, and radical scavenging activity. The study attempted to differentiate honey samples based on origin and composition. In the study, the Iranian honey samples were classified according to their respective physiochemical properties and antioxidant activities using principal component analysis and hierarchical cluster analysis. Furthermore, the relationships between the geographical and botanical origins were determined for the samples used in the study.

## Introduction

Honey is a widely-used sweet food, with a long history in human consumption. Honey is naturally-occurring, complex, and multi-functional, and is a good source of antioxidants. It is used in the preparation of foods and beverages, as well as medical and pharmaceutical purposes (1-3). At the present time, honey is required to meet specific quality criteria in order to be supplied in the market in most countries (1). Each of honey samples contains different compounds including oligosaccharides and other sugars, amino acids and proteins, elements, organic acids, odorants, phenolics and flavonoids, with various properties (4). Such complexity makes exact identification and classification of honey samples from different sources based on total composition practically impossible. Moreover, the high level of consumption of honey in many countries necessitates the adoption of comprehensive quality control measures. As such differentiating factors are assessed and screened in the present study for the purposes of classifying known honey samples and identifying test samples. The importance of honey sample authentication becomes even more apparent when considering countries or regions producing honeys from a diverse array of botanical and geographical origins. Currently, a proportion of the honey samples available on the market has been manipulated by low-quality ingredients through a process referred to as adulteration or has been described in misleading terms in relation to their geographical or botanical origins (5), while some others have been stored incorrectly. Due to such practices, the honey industry has given a considerable amount of attention to authentication practices and techniques throughout the world. Multivariate techniques have shown some factors in honey to be more representative in quality control and more discriminating in origin than others (1, 6-8). Geographical factors and botanical flora of the region can deeply affect certain properties, making it possible to use them to help determine the characteristics of the sample (4).

Multivariate techniques such as principal component analysis (PCA) and stepwise discriminant analysis can provide remarkable predictability with only certain features, which include electrical conductivity (EC), pH, free acidity (FA), percentage of fructose, glucose, and raffinose (1, 6-10).

Honey consumption is proven to play a role in the prevention of certain types of cancer (11), and is also believed to help reduce the risk of cardiovascular disease (12).

In the present study, the physicochemical properties and antioxidant activities of different Iranian honey samples are investigated using various multivariate techniques in order to develop a quality management model. Although some efforts have been made in recent years to develop a method for verifying the quality of honey samples, none have been able to provide an effective approach for the authentication of various Iranian honey samples. Investigation of particular Iranian local honey produced in the West Azerbaijan and Isfahan provinces, however, showed that their properties were in the normal range (13, 14). To the best of our knowledge, the present study represents the first comprehensive investigation of Iranian honey samples.

In this study, two different multivariate statistical techniques (CA, FA/PCA) were applied to data pertaining to physiochemical and chemical composition of honey samples that was obtained in the laboratory in order to evaluate Iranian honey from different botanical and geographical origins. To the best of our knowledge, this study is the first report on the classification of Iranian honey that uses multivariate statistical methods.

## Experimental

*Samples and reagents*

For this study, 48 honey samples of various botanical origins (17 different species) and from a range of different geographical regions (48 regions from 19/31 provinces) throughout Iran were donated by the Animal Sciences Research Institute (ASRI) in the summer of 2014. All reagents were analytical grade and were purchased from Merck Co. All spectrophotometric measurements were performed using a dual beam UV-Visible Cintra 101 series spectrophotometer, and all pH measurements were obtained using a Metrohm series 827 pH Lab.

*Botanical and geographical origins*

The samples were as follows: milk vetch (*Astragalus* spp.) (n = 11); thyme (*Thymus vulgaris* L.) (n = 12); christ’s thorn jujube (*Ziziphus spina-christi *(L.) Desf.) (n = 4); eucalyptus (*Eucalyptus *spp.) (n = 2); barberry (*Berberis vulgaris* L.); jujube (*Ziziphus jujuba* Mill.); manna of camelthorn (*Alhagi persarum* L.); coriander (*Coriandrum sativum *L.); acanthus (*Gundelia tournefortii* L.); sunflower (*Helianthus annuus* L.) (n = 2); dill (*Anethum graveolens *L.); parsley (*Petroselinum crispum* (Mill.) Fuss); alfalfa (*Medicago sativa* L.); cotton (*Gossypium hirsutum *L.); hami melon (*Cucumis melo* L. var. inodorus); pear honeydew (*Pyrus communis* L.); persian manna (*Astragalus adscendens *Boiss. and Hausskn.) (n = 5); and heated honey sample of persian manna (*Astragalus adscendens *Boiss. and Hausskn.).

**Figure 1:**
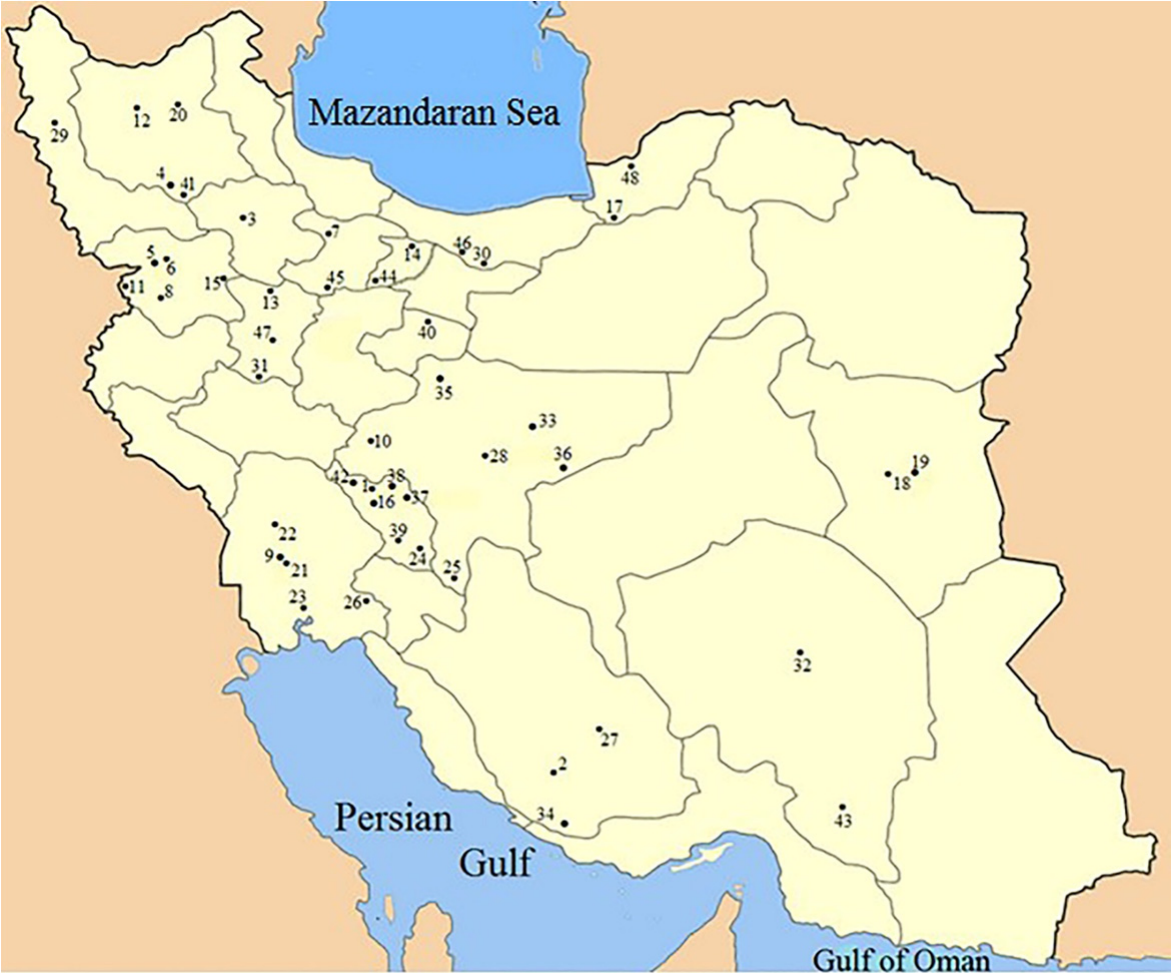
Distribution of samples through the country

Botanical origin of honey was first ascertained by melissopalynological analysis carried out by ASRI. Figure 1 locates the geographical origin of samples. Table 1 lists botanical and geographical origins of the honey according to their specified numbers on the map.

*Method*

 Fifteen parameters including physiochemical and antioxidant content indicators were measured/calculated as indicated below. All measurements were triplicated.

*Color intensity*

*ABS*_450_

 According to the method of Beretta *et al.* (2005), a 50% solution (w/v) was created by dissolving the honey sample in warm distilled water (45-50 °C). After filtration through a 0.45 µm filter at two wave lengths (450 nm and 720 nm), absorbance was measured and the difference between the values of absorbances was reported in milliabsorbance units (mAU) (15, 16).

*Refractive index and water percentage*

 The honey sample was homogenized and a portion of the homogenized sample was placed in an airtight flask in a thermostated water bath at 50 °C until all sugar crystals were dissolved. Then the refractive index (RI) of the sample was measured with an Abbe refractometer at a fixed temperature of 20 °C (n_20_). Water percentage (W%) was obtained from the table provided in the International Honey Commission (IHC) methods (17), or was calculated as follows (White, 1969):

 (1) W% = 400 (1.5380 – n_20_)

*Specific gravity and viscosity*

 These two parameters were obtained from the W% using the following formulas:

 (2) Specific gravity (SG) = -0.0063W + 1.5293

that was concluded from the data which present SG in different W% in 20°C.

(3) V = 530316 (e^-0.49w^)

like the above formula, was obtained from experimental data in which V is viscosity in poise and W is water content in percent (3).

*Electrical conductivity*

According to IHC methods, EC should be measured as follows: based on water content of honey, a 20% solution (W_dry matter_/V) is prepared in a thermostated water bath with a set temperature of 20 °C. EC was measured in milli-Siemens/centimeter (mS/cm) using a conductometer.

*Ash content*

There is a linear relationship between EC and ash content (AC) as follows:

(4) EC = 1.74 × AC + 0.14

where EC is electrical conductivity in mS/cm and AC is percentage of ash (3).

*pH and free acidity*

Ten g of the honey sample was diluted in 75 mL of carbon dioxide-free water. pH of the solution was measured with a digital pH-meter and FA was determined by titration up to pH 8.3 (17).

*Diastase activity*

Diastase activity was measured according to the Schade method presented in the harmonized methods of the IHC (17), with some modifications. In the current study, a 4% starch solution was used instead of a 2% solution, because, in the calibration process of starch solution to obtain optimum absorbance from different dilutions of starch, a 4% concentration proved more responsive. Therefore, to calculate the diastase number (DN), the calculation formula was multiplied by 2.

*Hydroxymethylfurfural (HMF)*

According to IHC methods, HMF is measurable in three ways (17). The basis of measurement of HMF concentration is its UV absorbance at 284 nm. HMF content of samples was measured using the White method.

*Proline*

The proline content could be readily determined by spectrophotometric comparison with a standard sample of proline after complex formation with ninhydrin as proposed by the harmonized methods of the IHC (17, 18).

*Total phenolic content*

Five mL of 10% water diluted samples was added to 2.5 mL of Folin-Ciocalteu 0.2 N solution. Folin-Ciocalteu reagent was used for the measurement of the total phenolic content (TPC) of the samples. The tubes were allowed to stand for 5 min, and then 2 mL of sodium carbonate (7.5%) were added and the test tubes were agitated. The absorbance of samples was measured after they had been standing for 2 h at room temperature at 760 nm (18). Gallic acid (10–1000 mg/L) was used as a standard to produce the calibration curve (R^2^ = 0.9985), and the results were expressed in mg of gallic acid equivalent (GAE) per 100 g of the honey sample.

*Antioxidant activity based on FRAP assay*

The total antioxidant capacity of the samples was determined using a ferric ion reducing antioxidant power (FRAP) assay (19).

*Radical scavenging activity and antioxidant content*

The scavenging activities of the honey samples were measured according to the methods of (18) with some modifications.

The calculations for AEAC values were expressed based on a curve obtained with a freshly prepared dilution series of ascorbic acid (0-100 mg/mL). The antioxidant content was expressed as mg of ascorbic acid per 100 g of honey.

*Analytical methods*

*Multivariate statistical methods*

Data were organized into a matrix of 48 rows (the honey samples) × 15 columns (composition). The results of the measurements were analyzed using various chemometric methods (20). Statistical methods were utilized to determine the mean and standard deviation of the measured variables and their mutual correlation coefficients. The chemometrics evaluation was performed using two multivariate techniques: factor analysis, including PCA, and cluster analysis, including hierarchical cluster analysis (HCA) using MATLAB 6.5 (TheMathworks, Natick, USA) and SPSS 11.5 for WINDOWS (SPSS Inc., Chicago, IL, USA, 2002).

*Data pre-treatment*

Data pretreatment methods were used to standardize the data. Among the methods used, auto-scaling of data has been selected. In this method, the mean of the corresponding column is first subtracted from each datum and then the results are divided by the standard deviation of the column. This method leaves each column with zero mean and unit variance.

*Factor analysis*

PCA was used to transform a two-dimensional multivariate data array into a new data set, so that some of the new variables (principal components (PCs)) could be used to explain most of the original data variability (21). PCA assumes that only a limited number of causes or sources (PCs) are responsible for most of the variance in the original data array.

The PCA technique starts with the covariance matrix describing the dispersion of the original variables (measured parameters), and extracting the eigenvalues and eigenvectors. An eigenvector is a list of coefficients (loading or weighting) by which the original correlated variables are multiplied to obtain new uncorrelated (orthogonal) variables, called PCs, which are weighted linear combinations of the original variables. A PC is the product of the original data and an eigenvector; the result of projecting the data onto a new axis is a new variable. There are as many PCs as original variables, but PC provides with minimal loss of information.

*Cluster analysis*

Cluster analysis is an unsupervised pattern recognition technique that uncovers the intrinsic structure or underlying behavior of a data set without making a priori assumptions about the data, in order to classify the objects of the system into categories or clusters based on their similarity. HCA is the most common approach by which clusters are formed sequentially. HCA starts with the most similar pair of objects and forms higher clusters step-by-step. 

The Euclidean distance usually gives the similarity between two samples, with the ‘distance’ being able to be represented by the ‘difference’ between analytical values from both of the samples. In HCA the process of forming and joining clusters is repeated until a single cluster containing all samples is obtained, yielding a result that can be displayed as a dendrogram, which provides a visual summary of the clustering process and presents a picture of the groups and their proximity with a dramatic reduction in dimensionality of the original data.

## Results

*Descriptive statistics*

In order to identify the main characteristics of Iranian honey samples, the statistics corresponding to the univariate data analysis of the measurements of 48 honey samples are shown in Table 2. Bivariate correlations between variables are shown in Table 3.

Approximately all ranges of parameters are subsets of the acceptable range (refer to national (according to recommendations of ASRI) and international standards (17)), with very few deviations. However, these results are derived from descriptive statistics, and applying statistical inference is necessary to achieve valid conclusions.

*Color intensity*

The color intensity of the honey samples ranged from 25 to 743 mAU. Accordingly, the color intensity of the all honey samples lies in the previously reported range (15). The lowest color intensity observed in the present study is in accordance with reported values. The highest number pertains to the hami melon of Buin Zahra (sample number 45) and is substantially greater than the next sample. The color of this sample is much darker than that of honey from other botanical origins, reflecting a tendency of the color of samples to become lighter with a decrease in their color intensities. In ABS_450_, measurements (mAU) varied from 25 (pale white) to 3413 (dark brown). According to the investigations of Beretta *et al.*, there is a strong positive correlation between the color intensity and the antioxidant properties of a honey sample. Marked differences in the color intensities of samples can serve as a reliable and accurate index of antioxidant activity (presence of carotenoids, flavonoids, Maillard reaction products). On the other hand, high color intensity can also result from specific contaminations arising during handling, processing, and storage, or from biochemical reactions that take place during maturation, yielding components with no antioxidant activity (15). Nocuous exposures or processes and differentiation between high antioxidant dark honey and low antioxidant dark honey can be detected by considering other honey quality criteria. AC of light-colored honeys is usually high. TPC and total flavonoid content can directly affect color intensity (22). Botanical origin of honey greatly affects honey composition, flavor, and color (23).

*Water percentage*

The optimum W% for honey is 18%. Our results show the greatest value of W% was approximately 17%. Among the honey samples examined, the smallest W% was 14.2%. After sugars, water is the primary component of honey. The W% of honey samples from West Azerbaijan province lies in the range of 13.97-15.87% (13). W% is the most important parameter related to honey quality. However, it is only of minor importance in the characterization of unifloral honeys. Usually honeydew honeys have lower W% than nectar honeys. There is reason to believe that honey processing can affect W% (24).

*Electrical conductivity*

ECs of samples change between 0.21 and 1.12 mS/cm and therefore are within the acceptable global range (0.06-2.17 mS/cm) (24). Three samples have EC values greater than 0.8 mS/cm (probably due to honeydew origin), nine samples have EC values between 0.51 and 0.79 mS/cm (mixing origin of honeydew and blossom), and the rest of the samples have EC values between 0.15 and 0.5 mS/cm (blossom origin) (5). A study of Indian commercial honey reported that each of the seven samples tested had EC values between 0.33 and 0.94 mS/cm (16). EC has a strong correlation with mineral content of honey, and it is usually used to determine the botanical origin of honey (4). In sugar-adulterated honey, some factors such as diastase activity, HMF content, AC, EC, and proline content are lowered. Determination of EC can be considered as the fastest method for routine honey quality control (5). EC was introduced in 1964 and at present is the most useful parameter for the classification of unifloral honey (25). EC values of several Spanish honey samples were reported by Mateo *et al.* as being in the range of 89 and 1213 µS/cm (6). In an investigation of Spanish honey, it was determined that EC and a_w _are the most discriminant variables, and that EC has more influence on discriminatory function and also has the highest discriminatory power (8). Mineral content can differ based on honeydew or nectar origin of honey, and EC can be used to determine kind and origin of honey (9).

*Ash content*

All samples used in the present study are in accordance with the standards of AC in honey used in the EU and Iran, which dictate that the ash percentage of honey cannot exceed 0.6%. ACs of the honey samples tested in the present study lie in the range of 0.041-0.562%. In comparison, in the study of 7 commercial Indian honey samples in 2010, ACs were reported as being between 0.03-0.43 g% (16). In a recent study of 6 honey samples from West Azerbaijan province in Iran, AC values obtained were between 0.01 and 0.49% (13).

Low EC and AC are typical of pale honey (26). The ease with which ash measurement can be carried out makes AC a useful parameter for differentiating between honey based on botanical origin, and for distinguishing between honeydew and nectar honey (9).

*pH*

All samples used in the present study have pH values greater than 3.5, and as such, are in accordance with the honey quality standards in Iran. The pH values of the samples tested range from a low of 3.62 to a high of 6.61. Considering global criteria (2); only three samples from the present study do not have pH values between 3.5 and 5.5. The 43 samples from the present study with pH values between 3.3 and 4.6 are most likely of blossom origin. Other more basic samples originate from honeydew. pH values in the study of French honey were between 3.46 and 6.48 (1). pH values of Uruguayan honey were between approximately 3.0 and 4.3. pH of honey is affected by conditions at the time of extraction and during storage, which also may affect stability, shelf-life, and texture. pH and EC have a high correlation (7). A combination of amino acid analysis, a_w _determination, sugar content analysis, pH value analysis, sensory evaluation, and statistical analysis could be the best method for ascertaining the botanical and geographical origins of honey (4). pH, FA, and total acidity have some limited applicability in the classification and discrimination of unifloral honey (25). In 1998, Spanish unifloral honey from seven different origins with pHs between 3.61 and 4.97 was classified based on physicochemical data (6).

*Free acidity*

The 45 samples that have FA values smaller than 40 mEq/kg are considered to be most desirable according to state standards. FA of honey samples from France was between 6.3 and 3.49 mEq/kg. A model only includes EC, pH, FA, and the percentage of fructose, glucose, and raffinose as variables classified with both the training and testing sets (1). In some studies, W% and FA were found to be the most important parameters for classification according to geographical origin by pattern recognition techniques applied to the chemical data (26).

*Diastase activity*

About 94% of the samples tested have DN values greater than 8. The 3 samples with DN values less than 8 still meet Iranian honey standards, as they are only required to have positive diastase activity. However, 2 of these 3 samples have DN values greater than 3. The DN values are between 1.9 and 55.2. Diastase analysis is one of the usual analytical methods for determinination of geographical or botanical origins of honey. This parameter is an indicator of honey freshness and heat treatment, and it is strongly affected by storage time and temperature conditions. Diastase activity is not legally allowed to be less than 8 DN units (1 DN unit hydrolyses 1 mL of 1% starch solution using 1 g of honey during 1 h at 37 °C). Since diastase activity varies by floral source, this criterion is not more accurate than the HMF content (maximum 40 mg/kg) (4). Some honey samples, such as citrus, acacia *etc.*, have naturally low enzyme content, a factor that must be taken into consideration when analyzing results (5). According to global standards, HMF and diastase limits are also valid after the honey has been processed and blended. According to an EU directive, HMF content, diastase activity, and honey acidity are considered to be applicable quality control standards for commercial partners and governments (5).

In comparison to previous investigations on honey samples from different regions of the world, the present study shows high DN values for Iranian honey samples. Devillers *et al.* (2004) reported that the DN values of 469 French honey samples from different botanical origins were between 8.56 and 38.56 (mean = 22.41) (1). In another study, 29 Spanish eucalyptus and citrus honeys were investigated, with the DN values obtained reported as being between 1.47 and 49.42 (mean = 19.3). Honey samples in that study were differentiated based on W%, HMF, diastase, pH, FA, lactone acidity, EC, glucose, fructose, sucrose, proline, invertase, glucose-oxidase, a_w,_ and insoluble solids (8). Based on a study by Saric *et al.* on a total of 254 Croatian samples between 2003 and 2005, DN was reported as being between 6.2 and 42.3 (9). Diastase activity of samples from the north and south of Cortha (Argentina) were 17.9 ± 6.7 and 20.3 ± 5.2, respectively (10). In a recent study on honey from West Azerbaijan province of Iran, DN values of 6 samples were reported as being between 9.41 and 25.2 (13).

*HMF content*

Thirty-two samples have HMF contents below 40 mg/kg, and the HMF contents of 3 samples from the tropics are each less than 80 mg/kg. Based on EU definitions, 14 honey samples can be labeled “virgin or quality honey”, because their HMF contents are below 15 mg/kg (3, 5). The HMF contents of 469 French honey samples were measured and reported as 3.287 ± 2.006 and were used along with other physicochemical parameters to classify honey. The aforementioned study confirmed that HMF does not participate in the formation of clusters (1).

Ideally, fresh honey should not contain HMF. Therefore, HMF should not be used as a criterion for the classification of samples. The importance of this parameter is in its utility in confirming freshness before determining storage-dependent parameters such as color and enzyme activity (25). Presence of HMF in honey depends on several factors, such as temperature, time of heating, storage conditions, pH, and floral origin (22). In the study of Uruguayan honey, HMF content fell in the range of 5.3-13.4 mg/kg and high correlations (r > 0.6) were observed between pH and HMF, HMF and EC. EC, pH, W%, and HMF proved effective in the classification of honey samples by floral origin, indicating that color is a less effective variable (7). In the study of two types of Andalusian unifloral honey, total HMF content was observed as being between 0.96 and 53.8 mg/kg, with significant correlations (*p*-value < 0.05) existing between invertase, diastase, and HMF (8). HMF content of Croatian honey was observed as lying between 0.4 and 99.8 mg/Kg (9). One of the reasons for the difference between HMF values of different honey, even in the same season, is variation in climatic conditions. The results of the study of the honey from northern and southern Argentina showed that HMF content for southern samples was higher than that for northern ones: 0.05-9.16 and 0.05-1.52 mg%, respectively (10). In the study of six varieties of floral honey from the West Azerbaijan province of Iran, HMF values of the honey varied from 0.04-17.2 mg/kg (13). Determination of HMF content is one means of detecting the addition of sweeteners and syrups used to replace the natural carbohydrates in honey. It should be noted, however; that HMF can be legally present in honey at levels up to 40 mg/kg, and in tropical or blend honey at levels up to 80 mg/kg. Therefore, investigating only one factor can yield ambiguous results, making it necessary to use various methods in combination with multivariate statistical techniques to determine the authenticity and botanical origin of the honey (27).

**Table 1:** The botanical origin of honey samples and their specific regions

**Sample Number**	**Botanical Origin, Geographical Region**	**Sample Number**	**Botanical Origin, Geographical Region**
1	Persian manna, Kuhrang	25	Milk vetch, Semirom
2	Christ's thorn jujub, Khonj	26	Christ's thorn jujub, Behbahan
3	Thym, Abhar	27	Thym, Neyriz
4	Thym, Takab	28	Manna of Camelthorn, Isfahan
5	Thym, Gardaneh Khan	29	Milk vetch, Urmia
6	Milk vetch, Gardaneh Khan	30	Thym, Yush
7	Milk vetch, Garab	31	Coriander, Nahavand
8	Milk vetch, Divandarreh	32	Acanthus, Kerman
9	Milk vetch, Dehdez	33	Sunflower, Isfahan
10	Persian manna, Fereydunshahr	34	Eucalyptus, South
11	Thym, Hawraman	35	Dill, Isfahan
12	Thym, Sabalan	36	Parsley, Isfahan
13	Thym, Razan	37	Persian manna, Kuhrang
14	Thym, Owrazan	38	Heated Persian manna, Kuhrang
15	Milk vetch, Sonqor	39	Milk vetch, Shahrekord
16	Persian Manna, Kuhrang	40	Alfalfa, Qom
17	Milk vetch, Gorgan	41	Thym, Takab
18	Barberry, Birjand	42	Persian manna, Kuhrang
19	Jujub, Birjand	43	Christ's thorn jujub, Jiroft
20	Milk Vetch, Sahand	44	Cotton, Eshtehard
21	Thym, Dehdez	45	Hami melon, Buin Zahra
22	Christ's thorn jujub, Shushtar	46	Thym, Sira
23	Eucalyptus, Ahvaz	47	Pear honeydew, Hamadan
24	Milk vetch, Gandoman	48	Sunflower, Hoseynabad

**Table 2:** Mean ± SD of physicochemical and antioxidant properties of samples

**Botanical Origin** **(Frequency)**	***Thymus vulgaris*** **(n = 12)**	***Astragalus *** **spp.** **(n = 11)**	***Astragalusadscendens*** **(n = 6)**	***Ziziphusspina-christi*** **(n = 4)**	***Eucalyptus *** **spp.** **(n = 2)**	**Helianthus annuus** **(n = 2)**	**Others** **(n = 11)**
ABS_450_ (mAU)	165.88 ± 57.05	129.26 ± 82.4	53.3 ± 16.3	250.99 ± 74.28	104.73 ± 14.94	180.82 ± 81.43	201.06 ± 196.2
RI	1.498 ± 0.002	1.497 ± 0.002	1.498 ± 0.0007	1.498 ± 0.002	1.5 ± 0	1.49 ± 0.0032	1.498 ± 0.002
W%	15.27 ± 0.73	15.59 ± 0.74	15.1 ± 0.15	14.8 ± 0.4	15 ± 0	15 ± 0	15 ± 0
SG	1.433 ± 0.004	1.431 ± 0.004	1.43 ± 0.001	1.43 ± 0.002	1.435 ± 0	1.435 ± 0	1.435 ± 0
Viscosity (poise)	313.96 ± 99.23	269.13 ± 84.21	325.1 ± 23.5	381.54 ± 81.82	340.64 ± 0.03	340.62 ± 0	340.63 ± 0.01
EC (mS/cm)	0.47 ± 0.18	0.38 ± 0.09	0.27 ± 0.03	0.67 ± 0.31	0.52 ± 0.15	0.45 ± 0.12	0.51 ± 0.24
AC (%)	0.19 ± 0.103	0.14 ± 0.05	0.075 ± 0.018	0.305 ± 0.18	0.22 ± 0.085	0.18 ± 0.072	0.21 ± 0.14
pH	3.95 ± 0.2	4.08 ± 0.4	4.02 ± 0.12	5.81 ± 1.17	4.06 ± 0.15	3.66 ± 0.06	4.07 ± 0.31
FA (mEq/kg)	30.67 ± 4.77	26.54 ± 6.87	21.83 ± 3.06	15.75 ± 3.09	39 ± 35.35	35.5 ± 4.95	27.18 ± 3.99
DN (Gothe units/g)	15.73 ± 5.85	14.77 ± 7.17	12.53 ± 2.44	28.68 ± 17.75	11.88 ± 0.05	16.68 ± 4.37	15.74 ± 5.45
HMF (mg/Kg)	56.76 ± 127.27	82.96 ± 136.59	30.7 ± 15.22	92.56 ± 175.45	35.33 ± 25.83	97.24 ± 129.1	93.35 ± 121.42
Proline (mg/Kg)	556 ± 153	492.5 ± 111	562.5 ± 105	495.5 ± 11	415.5 ± 225.5	414 ± 18.5	540.01 ± 141.32
TPC (mg GAE/100 g)	1076.73 ± 692.33	777.58 ± 469.72	461 ± 161.2	966.69 ± 271.41	512 ± 78.31	602.75 ± 80.96	845.43 ± 494.18
FRAP (mM Fe (II))	7.41 ± 2.96	5.58 ± 3.37	2.83 ± 0.38	4.93 ± 1.03	3.31 ± 1.28	4.38 ± 1.27	4.94 ± 2.105
IC_50 _(mg/mL)	20.4 ± 15.2	39.59 ± 36.07	88.425 ± 21.29	27.61 ± 18.34	71.38 ± 3.02	58.13 ± 31.18	45.63 ± 23.23

**Table 3:** Correlation matrix of honey samples variables (48 samples × 15 variable).

	**ABS**_450_	**RI**	**W%**	**SG**	**Viscosity**	**EC**	**AC**	**pH**	**FA**	**DN**	**HMF**	**Proline**	**TPC**	**FRAP**	**IC**_50_	
ABS_450_	1															
RI	-0.024	1														
W%	-0.075	-0.435	1													
SG	0.075	0.435	-1	1												
Viscosity	0.065	0.446	-0.979	0.979	1											
EC	0.446	0.074	-0.173	0.173	0.16	1										
AC	0.446	0.074	-0.173	0.173	0.16	1	1									
pH	0.295	0.178	-0.151	0.151	0.179	0.513	0.513	1								
FA	0.052	-0.076	0.045	-0.045	-0.059	-0.134	-0.134	-0.48	1							
DN	0.336	-0.096	-0.002	0.002	-0.009	0.248	0.248	0.194	-0.048	1						
HMF	0.139	-0.043	-0.135	0.135	0.107	-0.247	-0.247	-0.191	-0.019	-0.077	1					
Proline	0.125	0.223	0.04	-0.04	-0.048	0.137	0.137	0.037	0.334	0.181	-0.431	1				
TPC	0.658	0.054	-0.064	0.064	0.051	0.372	0.372	0.259	0.024	0.358	-0.066	0.139	1			
FRAP	0.574	-0.076	0.157	-0.157	-0.163	0.272	0.272	0.076	0.144	0.416	-0.108	0.215	0.88	1		
IC_50_	-0.535	-0.072	-0.13	0.13	0.092	-0.312	-0.312	-0.253	-0.095	-0.268	-0.009	-0.204	-0.627	-0.69	1	

**Figure 2:**
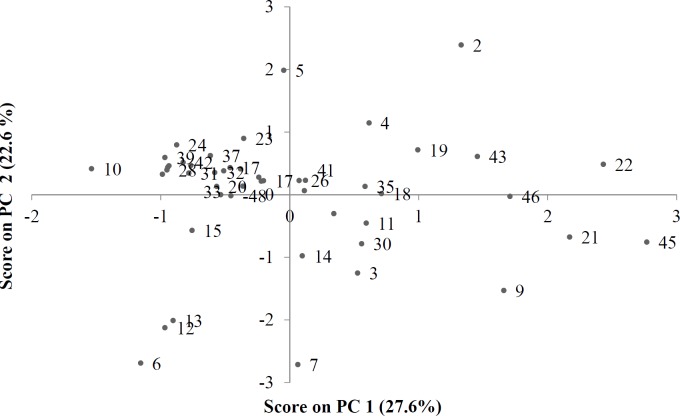
PCA score plot of honey samples from different botanical and geographical origins

**Figure 3.**
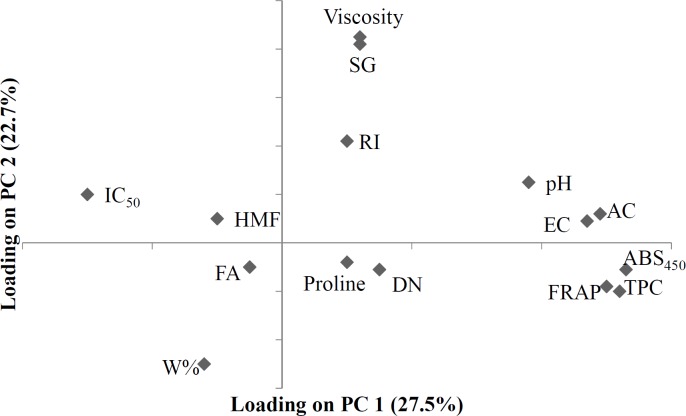
PCA loading plot of variables measured for physicochemical activity of honey samples

**Figure 4:**
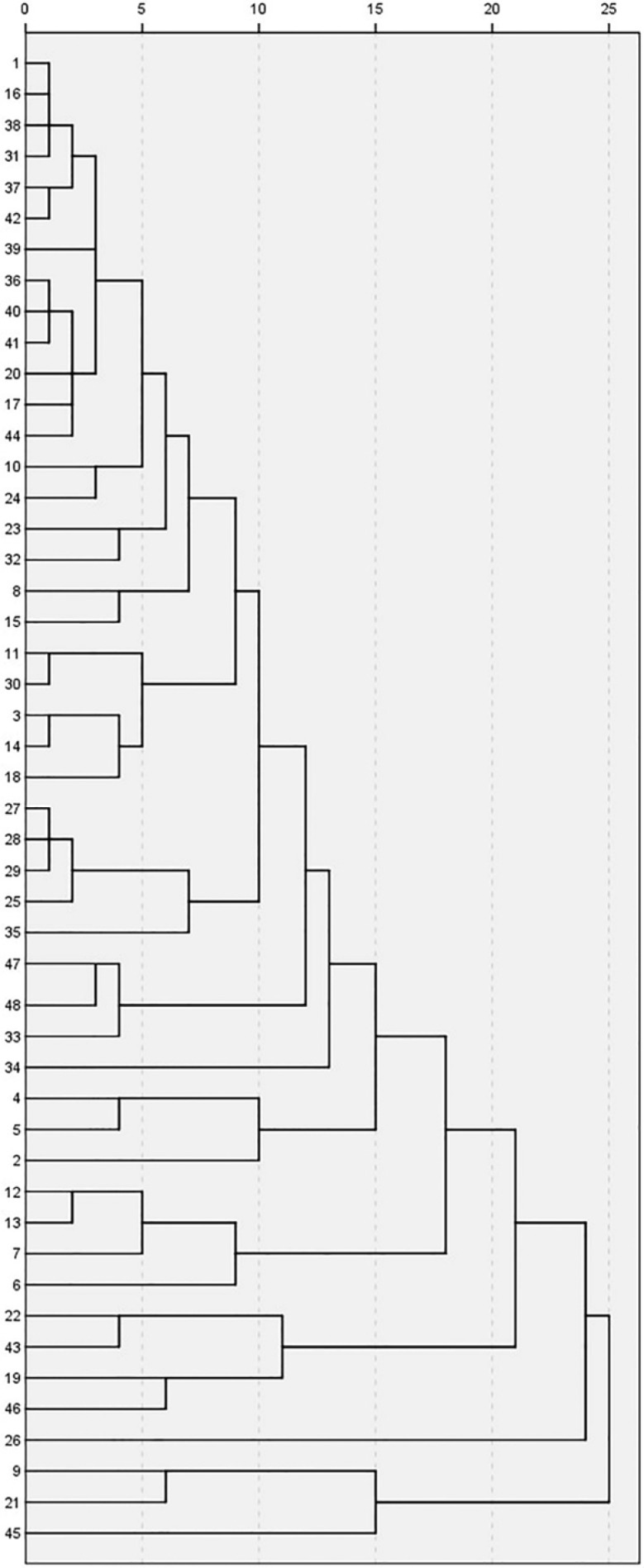
Dendrogram showing cluster analysis of different honey samples

**Figure 5:**
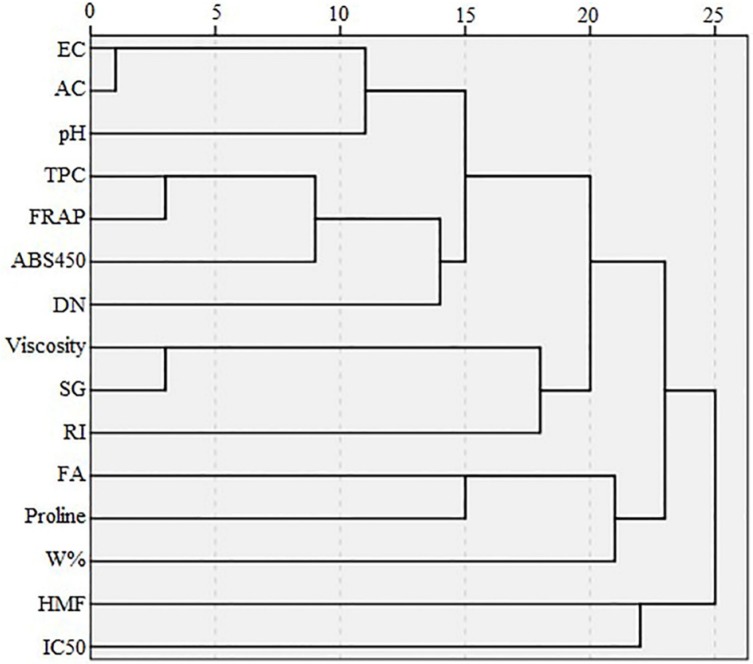
Dendrogram showing cluster analysis of different variables detected in honey samples

*Proline*

According to ASRI’s parameters for honey authentication, proline is not considered to be a routine quality control criterion. However, according to EU standards, the honey with proline contents below 183 mg/Kg is assumed to be adulterated (18). Proline content in the honey samples was found to vary from 240.4 to 848.07 mg/kg. Accordingly, one can assume that there has not been any sugar adulteration in these honey samples. In a study of Pakistani honey, proline content was found to be much lower than 183 mg/kg (28). According to the study of commercial Indian hones, proline content was found to be between 133 and 674 mg/kg. The correlation between proline with AEAC, DPPH scavenging, FRAP, phenol, and ABS_450_ was highly significant (16). From a quantitative point of view, proline is the most important amino acid and represents about 50% of the total number of amino acids present in honey, with its content having been reported as being higher in honeydew than in nectar honey (10). In the investigation of Croatian honey, proline content was reported between 24.0 and 1020.8 mg/kg (9). The proline content of two Andalusian honey samples from different botanical origins was reported as being between 36.95 and 986.63 mg/kg. The correlations between FA and proline; EC and fructose; and between proline, diastase, and sucrose are each significant (8). In sugar-adulterated honey, numerous chemical parameters, including proline content, enzyme activities, HMF content, AC, and EC were found to be outside of the acceptable range (5).

*Antioxidant activity*

Test results revealed that the TPCs were different from each other, a discrepancy that may be attributed to differences in the floral origins of the different types of honey. Dehdez honey (sample number 21) had the highest TPC (3020 mg GAE/100 g), while Fereydunshahr honey (sample number 10) had the lowest (193.8 mg GAE/100 g). A review of similar articles written about honey samples from other countries showed that the TPCs of the Iranian samples were high in comparison with those from other countries (16, 29). It seems that the floral origin of honey can affect the polyphenol profile of the samples (30). The same results were obtained from the radical scavenging reactions of honey samples with DPPH, as the IC_50_ value was 1.12% for Dehdez honey, which had the highest antioxidant activity, and 95.22 for Fereydunshahr honey, which had the lowest activity.

Assessment of the FRAP method was interpolated in a calibration curve of FeSO_4_.7H_2_O. As shown in Table 2, the results were expressed as mmol FeSO_4_/mL (R^2^ = 0.9866). Dehdez had the highest total antioxidant capacity with 14.84 mmol FeSO_4_/L, while Fereydunshahr honey had the lowest value with 2.34 mmol FeSO_4_/L (Table 2).

Further characterization of these honey samples using single variables was difficult to achieve, making it necessary to analyze data using multivariate techniques. The high correlations between the variable pairs are another reason for the application of the pattern recognition methods used in this study, and particularly PCA.

*Multivariate analysis of data*

*Principal component analysis*

PCA analysis showed that the first 5 PCs contain about 80% of the variance in data matrix. In other words, PCA can easily reduce dimension and complexity of a data matrix, while resulting in a loss of only about 20% of data. Similarities between honey samples were noticeable when PC1 was plotted against PC2 to obtain a score plot (Figure 2). Thus, all the Persian manna honey samples (numbers 1, 10, 16, 37, 38 and 42) are isolated in the left upper part of Figure 2, forming a strong cluster. This is also the case for the christ’s thorn jujube (numbers 2, 22, 26 and 43), which are all displayed in the right upper part of Figure 2. Sunflowers (numbers 33, 48) are displayed in the left middle part of Figure 2. Eucalyptus samples (numbers 23, 34) are shown in the middle, amenable to left and above. Thyme (numbers 3, 4, 5, 11, 12, 13, 14, 21, 27, 30, 41 and 46) and milk vetch (numbers 6, 7, 8, 9, 15, 17, 20, 24, 25, 29 and 39) samples appear together and alongside other samples. However, isolation can be seen as resulting from the climatologic or geographic origins of samples. Samples 9 and 21 are from Dehdez and are located in the lower right part of Figure 2. Samples 18 and 19 are from Birjand and are located close together. Samples 28, 33, 35, and 36 are all from Isfahan but they have different botanical origins.

In the loading plot (Figure 3), it can be observed that FA and pH appear far apart and in different zones from each other. In the loading plot, it can also be seen that, FA and pH are far from each other and are in different zones. The distance between these parameters in the loading plot reflects the fact that the greater the FA, the lower the pH. EC and AC are linearly and positively correlated, (AC was calculated from EC value by a linear equation) and were located at one point on the loading plot. Furthermore, EC and AC appeared very near to pH, which reflects their chemical relationship and their viability as a means of distinguishing unifloral honey samples (based on previous studies, EC and pH are very effective and precise in distinguishing unifloral honey). RI and W% have a negative, linear correlation. On the PCA loading plot these two variables are very far apart and in different zones. Viscosity and SG, which were calculated from W% by their relevant equations, were shown to have a negative correlation with W% and were located at one point far from W% and very near to RI. Based on the loading plot, it can be observed that W% is a unique parameter that is located very far from every other factor, making it a viable factor for use in honey quality control and authentication.

The first PC shows the highest positive correlations with color, EC, AC, Folin and FRAP, and the highest negative correlation with IC_50_, and extracts 28% of the variance of data matrix. The second PC shows a strong positive correlation with SG and viscosity, and a strong negative correlation with W%. The second PC extracts 22.7% of the data variance. When score and loading plots are superimposed, it is evident that the variables are most effective in each PC. As can be seen in Figures 2 and 3, it seems that HMF and IC_50_ have the most important effects on the close proximity of the 6 Persian manna honey samples on the plot. Four Christ’s thorn jujube honey samples are concentrated in a small region, due to the effect of viscosity, SG, RI, pH, EC, and AC. Two sunflower honey samples are brought close together by HMF and FA. The two eucalyptus samples appear close to each other due to the effect of RI, HMF, and IC_50_. Two Dehdez’s samples are near together due to the effect of color intensity and antioxidant properties. Honey samples of Birjand appear close to each other due to the effect of physical properties. Isfahan’s honey samples appear near each other due to the effect of RI, HMF, and IC_50_.

The results of the PCA show that correlating factors generally verify each other: the greater the color intensity by ABS_450_, the greater the antioxidants properties by folin, FRAP, and proline, but the smaller IC_50_. High DN and low HMF content indicates honey freshness, and according to the loading plot (Figure 3), these two variables show a negative correlation. The correlation matrix (Table 3) and obtained values of the parameters examined (most honey has high DN and low HMF content) can be used to verify the results of the PCA. DN could be used as a relative indicator for originality of the honey because the honey bee produces the enzyme; therefore in Figure 3, DN is very close to proline.

*Hierarchical cluster analysis*

In the present study, HCA is used to show the natural groupings within the dataset. This method is especially effective when the number of the cases examined is limited. Ward’s method, which uses square Euclidean distances, was used to show the linkages between similar samples. In the present study, we used this technique in both samples and variables, and compared the results with those obtained from PCA. For this reason, the dendrogram was employed as a graphical tool to show the clusters. In Figure 4 honey samples are listed along the left vertical axis. The horizontal axis shows the distance between clusters when they are joined. Dividing the dendrogram to determine the number of clusters is a subjective process. Generally, we begin by looking for “gaps” between joints along the horizontal axis by observation from right to left. It is easy to see in Figure 4 that these clusters correspond to specific honey types.

There is a gap between 20 and 25 that divides the honey samples into two clusters: three samples in one cluster and the remainder in another one. There is a subdivision in the bottom cluster that places the honey sample number 45 as a unique sample in one cluster and the other two samples (9 and 21), which have the same geographical origin, in another cluster. According to the score plot of PCA (Figure 2), sample 45 is near the margin of the plot and far from almost all of the other samples. Samples 9 and 21 on this plot appear near sample 45. There is another gap between samples 15 and 20, which divides the other 45 samples into two clusters. There is only one sample (26) in the lower cluster. By continuing the investigation of the dendrogram, another gap is observed between samples 10 and 15, which divides the 44 samples into two clusters. The lower cluster contains four samples from different locations, but the two samples (22 and 43) between them have the same botanical origin and were placed in a separate cluster, which is a subdivision from the previous cluster. Another sample (19) is from a different botanical and geographical origin, but the latitude, climate, and probable soil composition of its origin are similar to those of samples 22 and 43. Score and loading plots of PCA show that these four samples (19, 22, 43, and 46) are very similar with regards to EC, pH, color, and DN.

The dendrogram of variables (Figure 5) shows that HMF and IC_50_ are in the same cluster, similar to the results of loading plot of PCA (Figure 3). Proline, FA, and W% are in the same cluster, but W% is located in a different cluster after subdivision of the previous cluster. Viscosity and SG are in the same cluster and their cluster with RI forms an additional cluster. Similar to PCA results, folin and FRAP have a strong correlation and appear in the same cluster which in turn forms another cluster with color intensity. An additional cluster is formed between the three aforementioned variables and DN. Based on the PCA results, EC and AC appear at the same point on the loading plot, and in one cluster in the dendrogram. On the loading plot of the PCA, pH appears close to EC and AC; pH similarly forms a cluster with the cluster of EC and AC in the dendrogram.

The honey samples from different botanical origins, but the same climatic or geographic region, may have the same or similar compositions, and cannot be separated via classification analyses. Furthermore, there are not huge farmlands in Iran, which leads honey bees to be exposed to several herbs in each territory and to collect different nectars. It seems that gathering of monofloral honey is not a common practice. The fact becomes more significant when considering the transportation of the hives between different regions during different seasons by beekeepers. Beekeepers usually blend gathered honey products from hives located in different regions.

The results of chemometric classification methods carried out on physicochemical and antioxidant properties are highly matched to melissipalynological investigations performed by ASRI. In other word, honey samples of similar botanical or geographical origins were clustered together or near each other in most cases.

## Discussion

From the perspective of healthcare, finding a means of differentiating natural from handmade, adulterated, or misrepresented honey is extremely important. For instance, while the ability of natural honey to increase serum HDL-C level gives it strong cardio-protective properties, handmade honey has been shown to decrease such levels (31). Determination of enzymatic activity in various honey samples could be used to classify their antimicrobial and wound healing effects, meanwhile such enzymatic activity along with others implies storage period, heat exposure, and the inherent quality (5, 32 and 33). Some physicochemical properties such as pH, color, W%, EC, *etc.* can vary among different honey samples (6).

Besides the physicochemical and antioxidant properties discussed above, some other properties like Cu, Mn, and Li content, as well as rheological properties have been used for chemometric classification of honey samples (15, 16, 22, 34-37).

Authentication and adulteration-detection techniques can be developed by assessing physiochemical factors and comparing them with defined standards or previously accepted measurements (5, 24).

Biomimetic sensors (electronic tongue, electronic nose) paired with neural network analyses can be used to facilitate the detection of sugar adulteration in the honey, since laboratory assessments are not capable of detecting this type of adulteration (38).

For religious and cultural reasons, Iranian people have traditionally regarded honey as being both highly valuable and nutritious, and even as having healing properties. As a result, the high demand and price of honey (3 to 10 USD per kg) creates an incentive for adulteration. Iran’s production and per capita consumption of honey have increased threefold over the past two decades, and the country’s per capita consumption is twice that of the rest of world (38). There are currently approximately 65,458 beekeepers with a total of 5,613,259 beehives in Iran. Average production of honey/beehive/year is 13 kg. Accordingly, 71,142 tons of honey is produced in Iran annually, 3,700 tons of which are exported (39). As such, the development of quality control and pattern recognition techniques for the authentication of Iranian honey can be understood to have significance throughout the globe, particularly for countries that import Iranian honey. This study shows that honey from different regions of Iran meet high quality criteria and additionally labeling of honeys based on their botanical and geographical origins within Iran is largely accurate.

Multivariate analysis techniques are very helpful for the discrimination and classification of the samples according to their origins, because some processes like pasteurization have no effect on some factors (HMF and diastase, in the case of pasteurization) while producing a negative effect on some others (invertase, in this case).

Chemometric combination of different factors including W%, proline, AC, EC, acidity, pH, HMF, diastase, and sugars leads to a strong differentiation of honey from different geographical regions of Spain (5).

Thus, simultaneous assessment of physico-chemical factors can be discriminant. There are two common methods of unsupervised pattern recognition, namely cluster analysis (CA) and PCA with factor analysis. The results of CA help in the interpretation of the data and in pattern recognition. Factor analysis, including PCA, is a powerful tool, which is used to identify correlations among numerous measurement variables and samples. The full data set is described in a reduced number of PCs, with no significant loss of information (40).

Many examples of the application of this type of statistical analysis can be found elsewhere. Ruoff *et al.* reported that physico-chemical analysis cannot be used to authenticate the honey samples, but that chemometric evaluation of these factors by linear discriminant analysis can be used to authenticate honey samples without the use of pollen or sensory analysis (41).

## Conclusion

Evaluation of the effect of the various methods and the botanical origins on the physicochemical and antioxidant content of Iranian honey with the aid of chemometrics as a powerful classification method was not carried out due to the lack of any previous comprehensive study on the subject. A good approach would be to perform several different types of cluster analysis and to compare the results obtained from those analyses. In the present study, we were able to classify some of the Iranian honey samples according to their respective physicochemical and antioxidant properties, and to show both the relationships between the botanical and geographical sources and the properties of those samples from which the samples were obtained by chemometrics.
